# [Retracted] MicroRNA‑219 is downregulated in non‑small cell lung cancer and inhibits cell growth and metastasis by targeting HMGA2

**DOI:** 10.3892/mmr.2024.13237

**Published:** 2024-05-08

**Authors:** Xiaoping Sun, Min Xu, Haiyan Liu, Kunxiu Ming

Mol Med Rep 16: 3557–3564, 2017; DOI: 10.3892/mmr.2017.7000

Following the publication of this paper, it was drawn to the Editor's attention by a concerned reader that certain of the Transwell migration and invasion assay data shown in Figs. 2C and 4C were strikingly similar to data that had already been published in different form in another article written by different authors at a different research institute [Yang S, Zhang Y, Zhao X, Wang J and Shang J: microRNA-361 targets Wilms’ tumor 1 to inhibit the growth, migration and invasion of non-small-cell lung cancer cells. Mol Med Rep 14: 5415–5421, 2016].

Owing to the fact that the contentious data in the above article had already been published prior to its submission to *Molecular Medicine Reports*, the Editor has decided that this paper should be retracted from the Journal. The authors were asked for an explanation to account for these concerns, but the Editorial Office did not receive a reply. The Editor apologizes to the readership for any inconvenience caused.

